# Concept of the Number Needed to Treat for the Analysis of Pain Relief Outcomes in Patients Treated with Spinal Cord Stimulation

**DOI:** 10.3390/biomedicines10020497

**Published:** 2022-02-20

**Authors:** Ashley Bailey-Classen, Amar Parikh, Nima Adimi, Deborah Edgar, Alice Yan, Anand Rotte, David Caraway

**Affiliations:** 1 Trinity Pain Medicine Associates, Fort Worth, TX 76102, USA; ashleybaileyclassen@gmail.com; 2 OrthoNY Spine and Back, Albany, NY 12205, USA; aparikh22@gmail.com; 3 Ridgeview Spine and Pain Center, Chaska, MN 55318, USA; nima.adimi@ridgeviewmedical.org; 4 Commexus Ltd., Dunblane FK15 0DF, UK; dedgar@commexus.com; 5 Nevro Corp., Redwood City, CA 94065, USA; alice.m.yan@gmail.com (A.Y.); anand.rotte@gmail.com (A.R.)

**Keywords:** NNT, SCS, pain relief, back pain

## Abstract

In the rapidly evolving field of spinal cord stimulation (SCS), measures of treatment effects are needed to help understand the benefits of new therapies. The present article elaborates the number needed to treat (NNT) concept and applies it to the SCS field. We reviewed the basic theory of the NNT, its calculation method, and its application to historical controlled trials of SCS. We searched the literature for controlled studies with ≥20 implanted SCS patients with chronic axial back and/or leg pain followed for ≥3 months and a reported responder rate defined as ≥50% pain relief. Relevant data necessary to estimate the NNT were extracted from the included articles. In total, 12 of 1616 records were eligible for inclusion. The records reported 10 clinical studies, including 7 randomized controlled trials, 2 randomized crossover trials, and 1 controlled cohort study. The studies investigated traditional SCS and more recently developed SCS modalities, including 10 kHz SCS. In conclusion, the NNT estimate may help SCS stakeholders better understand the effect size difference between compared treatments; however, interpretation of any NNT should take into account its full context. In addition, comparisons across trials of different therapies should be avoided since they are prone to interpretation biases.

## 1. Introduction

Chronic pain is a common problem among adults, with prevalence studies suggesting that one in five are affected [1,2]. Despite considerable advances in our understanding, diagnosis, and management of pain, many patients report inadequate pain control [2,3,4,5]. Effective pain management strategies are needed to address unrelieved pain among this population.

Spinal cord stimulation (SCS) has been used for over 50 years to treat various refractory chronic pain syndromes. During SCS, an implanted device delivers low-level electrical pulses to the spinal cord via an array of electrodes placed into the epidural space either percutaneously or surgically via laminotomy or laminectomy. In traditional SCS (t-SCS), perceptible paresthesia is elicited over the area of pain by individual pulses delivered continuously at a fixed low frequency (40–50 Hz), pulse width (150–500 µs), and amplitude [6].

Over the last decade, SCS technology has developed rapidly, resulting in the emergence and adoption of several new stimulation paradigms, including high-frequency 10 kHz SCS (10 kHz SCS), burst stimulation, dorsal root ganglion (DRG) stimulation, and closed-loop SCS [7,8,9,10,11,12,13,14,15]. Never has more choice been available in the field of SCS since the inception of the technology. Appropriate application of these new stimulation paradigms may offer clinical benefits to patients. However, with so many treatment options to consider, readily understandable measures of treatment effect are needed.

First described over 30 years ago as a way of summarizing the binary outcomes of randomized controlled trials (RCTs) [16], the number needed to treat (NNT) may be one such measure. When comparing two treatments, the NNT tells us how many patients need to be treated with one treatment instead of the other for a given time before we expect one extra patient to achieve a positive outcome of interest (or avoid a negative one) [17,18,19]. The clinical trial reporting guidelines (CONSORT) and the British Medical Journal (in its instructions to authors) suggest reporting the NNT where possible [20,21]. Systematic reviews and meta-analyses have also presented the NNT [22,23]. The popularity of the measure reflects its intuitiveness and ability to communicate the results of controlled trials effectively. However, despite widespread reporting in other medical disciplines, the NNT has not been widely or accurately adopted in the field of SCS. 

Since the metric could be a useful tool to aid clinical decision-making, this article aims to elaborate the NNT concept and apply it to the SCS field. We provide an overview of the definition, calculation, and interpretation of the NNT, as well as an evaluation of the metric for historical controlled trials of the therapy in chronic axial back and/or leg pain patients.

## 2. Materials and Methods

### 2.1. Calculation and Interpretation of the NNT

Randomized controlled trials typically compare a treatment with a control arm receiving either a different treatment, placebo, or other appropriate control. Analysis of a binary endpoint usually yields a proportion of patients in each group with a positive outcome of interest [17]. The difference between the proportion of patients with the corresponding negative outcome in the control arm and treatment group is called the absolute risk reduction (ARR) [17]. It reflects the proportion of the population that is spared the negative outcome [24]. The NNT is the reciprocal of the ARR [17,19,25]. For example, consider a trial of a new therapy’s effectiveness versus standard treatment, which reports the responder rate in each group as 50% and 30% at the 12-month follow-up, respectively. In this case, the corresponding negative outcome is nonresponse to treatment, with rates of 50% and 70%, respectively, yielding an ARR of 20% and an NNT of 5. The NNT of 5 means that if we treat 5 patients with the new therapy instead of the standard treatment for 12 months, we would expect to see one extra responder.

Over and above its simplicity, the NNT is often cited as a useful measure because it reflects absolute rather than relative risk reduction [26,27]. The best possible NNT is 1, which means all patients are likely to respond to the new treatment and none to the comparator treatment. Generally, the closer the NNT is to 1, the more effective the new treatment is versus the comparator treatment. If the difference between the therapies is small, the NNT will be high, up to a maximum of infinity (i.e., equal responder rates or zero response in both groups). Although single-digit NNTs are normally desirable, higher NNTs can be acceptable if the outcome of interest is the prevention of a serious outcome [18,24]. A negative NNT indicates that the comparator treatment had better outcomes than the new treatment [24].

The correct interpretation of the NNT for a therapy relies upon understanding its context, including the comparator treatment, baseline risk of the patients, outcome measure, and time frame [28]. A change in any of these factors will yield a different NNT. Since comparisons across trials of different treatments are prone to interpretation biases, it is also important that an NNT analysis specifies how the value was derived and any limitations associated with the process. Furthermore, it is also critical to understand whether the difference in outcome between the two treatments is statistically significant. Typically, this information is conveyed by a 95% confidence interval (CI) around the NNT estimate, constructed by inverting and reversing the limits of the ARR 95% CI [17,24].

### 2.2. NNT Calculation

The NNT and its 95% CI are calculated using standard methods [17,25,29,30,31]:

Defined notations:

Number of nonresponders in the treatment group: *a.*

Number of responders in the treatment group: *b.*

Number of nonresponders in the control group: *c*.

Number of responders in the control group: *d.*

Total number of subjects in the treatment group: N_T_.

Total number of subjects in the control group: N_C_.

Absolute risk of nonresponse in the treatment group: $ART=\frac{a}{a+b}$ .

Absolute risk of nonresponse in the control group: $ARC=\frac{c}{c+d}$ .

Absolute risk reduction: ARR=ARC−ART .

*ARR* standard error (SE) of the mean (normal approximation):

$\mathrm{SE}\left(ARR\right)=\sqrt{\frac{ARC\left(1-ARC\right)}{{\;N}_{C}}+\frac{ART\left(1-ART\right)}{N_{T}}}$ .

*ARR* 95% CI (Wald method): 95% CI (ARR)=(ARR−1.96×SE(ARR),ARR+1.96×SE(ARR) ).

Number needed to treat: $NNT=\frac{1}{ARR}$ .

*NNT* 95% CI.

For a statistically significant treatment difference (i.e., *ARR* 95% CI does not span zero): $95\%\;\mathrm{CI}\;\left(NNT\right)=\left(\frac{1}{ARR+1.96\times \;\mathrm{SE}\left(ARR\right))},\frac{1}{ARR-1.96\times \;\mathrm{SE}\left(ARR\right))}\right)$ .

For a statistically nonsignificant treatment difference (i.e., *ARR* 95% CI spans zero): 

$95\%\;\mathrm{CI}\;\left(NTT\right)=\left(\frac{1}{ARR-1.96\times \;\mathrm{SE}\left(ARR\right)},\frac{1}{ARR+1.96\times \;\mathrm{SE}\left(ARR\right)}\right)\;$ ^†^.

^†^ Discontinuous CI: (−∞ to lower boundary) ∪ (upper boundary to ∞).

### 2.3. SCS Literature Review

#### 2.3.1. Data Sources

This literature review focuses on the responder rate results of prospective controlled studies evaluating SCS in subjects with chronic axial back and/or leg pain. Clinical trial information was accessed by online search using the PubMed resource and ClinicalTrials.gov registry. Websites of SCS suppliers were also reviewed for relevant publications. No date constraints were applied to the searches.

The PubMed and ClinicalTrials.gov (accessed on 3 October 2021) search strategies utilized search terms relevant to SCS and axial back and/or leg pain, including the following: spinal cord stimulation, dorsal column stimulation, epidural stimulation, low frequency stimulation, high frequency stimulation, HF10, 10 kHz, burst stimulation, dorsal root ganglion stimulation, DRG stimulation, chronic pain of the trunk and/or limbs, back pain, spinal pain, post laminectomy syndrome, post laminectomy pain, failed back surgery syndrome, failed back surgery syndrome (FBSS), axial back pain, axial pain, sciatica, leg pain, radicular pain, lower limb pain, and lower extremity pain.

#### 2.3.2. Eligibility Criteria

Records identified during searches were considered eligible for inclusion if they reported clinical trials that fulfilled the following criteria: prospective controlled design; at least 20 patients treated with a permanent SCS implant and followed for 3 months or more; at least 80% of the population suffering from a primary complaint of chronic axial back and/or leg pain (e.g., due to FBSS, radiculopathy, disc degeneration, or lumbar stenosis); and responder rate efficacy outcome reported (and defined as at least 50% pain relief from baseline based on a numerical pain rating scale or visual analog scale). Articles were excluded if the reported study evaluated an SCS system delivering multiple SCS modalities, a retrospective control group, or different technical aspects of an SCS system (e.g., percutaneous leads versus surgically placed electrodes). In addition, all conference proceedings, non-English articles, reviews, case reports, letters, and editorials were rejected. Where information was insufficient, the reviewer performed a full-text evaluation.

#### 2.3.3. Data Extraction

Full-text articles were retrieved where possible for all records that met the eligibility criteria. Extracted data elements included: author information, publication year, study name, study design, pain relief measure, SCS trial information, SCS indication, SCS stimulation modality, proportion of patients with axial back and/or leg pain, proportion of patients with FBSS, follow-up time, and responder definition. We also documented the responder rate, total number of subjects, number of responders, and number of nonresponders per pain area and follow-up for both the intention-to-treat (ITT) and per-protocol (PP) populations. If necessary, the number of responders and nonresponders were calculated from the responder rate and group sample size. In addition, the same outcome data were documented for comparative treatments.

## 3. NNT Applied to SCS Controlled Trials

The NNT was estimated for the SCS controlled trials using within-trial data. The ITT population was defined as (1) the number of subjects randomized or assigned to the treatment and control groups at the start of the study or (2) the number of subjects included in an interim analysis of a partial study population. The PP population was defined as the number of subjects in each group with available data at the specified follow-up. The standard calculation method outlined above was used to estimate the NNT per follow-up time and pain area for the ITT and PP populations.

## 4. Results

### 4.1. Literature Review Results

**Figure 1:**
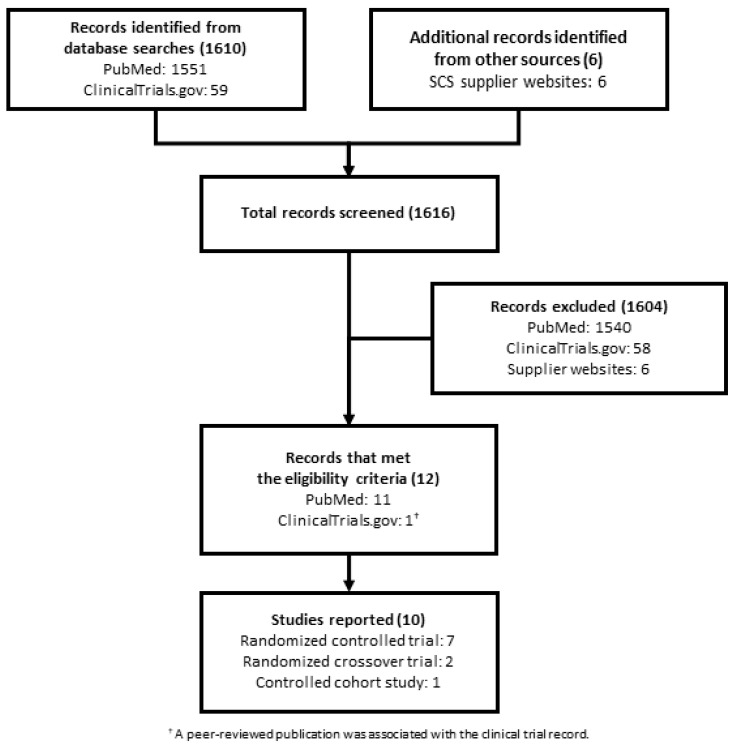
Literature search flow chart.

Among 1616 records identified in the PubMed, ClinicalTrial.gov, and SCS suppliers website searches, 1604 failed screening (Figure 1). The remaining 12 records that met the eligibility criteria reported 10 clinical studies and comprised 12 full-text articles.

Summary characteristics of the 10 included studies are presented in Table 1. The study designs included seven RCTs [30,32,33,34,35,36,37,38,39], two randomized crossover trials (RCOTs) [40,41], and one controlled cohort study [42]. Types of stimulation investigated in the studies comprised both traditional [30,32,33,37,42] and more recently developed SCS modalities, including ≤1.2 kHz subperception SCS [41], 10 kHz SCS [34,35], burst stimulation [40], closed-loop SCS [38], externally powered 10 kHz SCS [36], and differential target multiplexed SCS (DTM-SCS) [39]. While at least 80% of all patient populations were deemed to have chronic axial back and/or leg pain, the proportion with previous surgery (i.e., FBSS diagnosis) ranged from 42% to 100%. The SCS indication varied across the studies according to whether both back and leg pain was present and if one pain area was dominant over the other. Several studies included additional elements in their response definition over and above at least 50% pain relief from baseline [32,34,35,38,41].

### 4.2. NNT Estimates for SCS Controlled Trials

Ideally, the NNT should be estimated within well-designed controlled trials. Our literature search identified 10 such trials for the treatment of chronic axial back and/or leg pain [30,32,33,34,35,36,37,38,39,40,41,42]. The estimated NNT values for both the ITT and PP populations in these trials are summarized in Table 2 (calculated from data presented in Table S1—see Supplementary Information). For each population, the NNT estimates are specific to the therapies evaluated, baseline risk, response definition, pain area, and follow-up time.

## 5. Discussion

In the past, controlled trials of SCS therapy have generally focused on inferential statistical analyses, which provide information about the statistical significance of group differences. However, *p* values tell us little about the clinical significance of the results, i.e., the magnitude of the treatment effect [43]. One widely used measure of treatment effect is the NNT. The present article applies the concept of the NNT to the SCS field and explains its interpretation in the context of pain relief. To our knowledge, this is the first presentation of NNT estimates for historical trials of SCS. This topic is particularly relevant given the remarkable advancements in the field over the last decade, including the introduction of several new stimulation waveforms, which may improve clinical efficacy and expand indications. The pace of technological innovation is likely to increase, bringing with it ever-increasing choice and the need for easily interpretable measures of treatment effect.

Our search of the SCS literature identified a small number of controlled trials with responder rate data that compared one type of SCS stimulation with another in patients with axial back and/or leg pain. Types of stimulation investigated in the studies comprised both traditional and more recently developed SCS modalities. From this literature, we estimated the NNT for the controlled trial populations.

The NNT statistic can help clinicians understand the clinical relevance of binary outcomes from an individual comparator trial. It may also be used to inform cost-effectiveness analyses (CEAs). A review of CEA studies that incorporated the NNT suggested that such studies had a high clinical impact since they were generally published in clinical practice-focused journals [44]. The majority (>90%) of respondents in an international survey of policymakers and other stakeholders also considered that the magnitude of treatment effect is an important criterion in health system decisions [45]. In addition, all of the survey respondents considered the inclusion of this information important or probably important.

## 6. NNT Interpretation

Number needed to treat estimates can range from 1 to ∞, although they can be negative in specific circumstances. A perfect NNT would be 1, meaning that all patients will likely respond to the new treatment and none to the comparator treatment. In practice, this is an unlikely outcome. However, the closer the NNT is to 1, the greater the effect size difference between the treatments [46]. As the NNT increases, the effect size difference diminishes until NNT reaches ∞, indicating no difference between the two treatments or zero response in both study arms. As a general rule of thumb, an NNT below 10 may be considered clinically meaningful in the right context since one additional positive outcome would be encountered relatively often in everyday clinical practice [46]. However, high NNT estimates may be acceptable if the outcome of interest is the prevention of a serious event such as myocardial infarction or stroke, or if the condition is difficult to treat and other therapy options have failed or are very limited [18,24]. If a comparator treatment had better outcomes than a new treatment, the NNT would be negative [24].

Although the number needed to treat statistic is popular, it should be interpreted with caution. The phrase can easily be misread as implying the number needed to treat to produce one positive outcome of interest. However, in a given time frame, the NNT only estimates how many patients need to be treated with one treatment instead of another before we expect one extra patient to achieve a positive outcome of interest. The NNT does not guarantee that an extra positive outcome will occur, nor does it predict which patient may benefit; it only gives an expected value [19]. Understanding the context of the NNT is essential to its interpretation. It is also important to describe how the calculation was derived along with its limitations since comparisons across studies of different treatments are prone to interpretation biases. The NNT context includes several key factors, including the following.

### 6.1. Comparator Treatment

Given the utility of NNT, it is tempting to look at this metric across RCTs and compare, perhaps considering the lowest number as the “best” treatment. However, this is not always a valid approach. For example, the comparator treatment for each study must be known [28]. In SCS, a new modality is typically compared with traditional SCS or conventional medical management (CMM). Each of these comparisons will yield a different NNT. A new active therapy applied in the control arm (such as a different form of SCS) would be expected to generate a higher NNT than for the same test treatment compared to an ongoing failed treatment such as in CMM-controlled trials. Comparator treatment nuances are also worthy of examination, especially in multicenter studies where the application of a treatment may vary between clinics and geographies, for example, SCS programming or usual clinical care. In pharmaceutical trials, NNT estimates are often, appropriately, compared across therapies when the comparators are all placebo and the populations tested are similar [22].

### 6.2. Baseline Risk

The NNT metric is inversely related to baseline risk [28]. In addition, patients with different baseline characteristics may respond differently to treatment and produce variable NNT estimates [47]. For example, among the studies identified in our review, the proportion of subjects with previous spinal surgery varied widely, from 42% to 100%. In addition, several studies recruited patients with predominant leg pain [30,32,33,42], while other populations had back and leg pain [34,35,38,39], back pain with or without leg pain [36], predominant back pain [37], or trunk and/or limb pain [40,41]. Other baseline characteristics may also influence how patients respond to therapy. An example among the studies in our review is the Turner et al. (2010) study that recruited workers’ compensation recipients [42]. Patients treated under compensation schemes may respond less well to pain therapy [48,49,50], including SCS [51], than uncompensated patients. Being aware of such characteristics aids the understanding of NNT estimates that may appear anomalous.

### 6.3. Time Frame

The time at which the treatment outcome is measured must also be considered [28,52]. For example, an NNT calculated at 3 months of follow-up could differ from that assessed at 12 months. Treatment efficacy may also take time to accrue, and negative treatment effects may resolve over time [18]. 

### 6.4. Outcome Variable

Another critical aspect of the NNT is the outcome variable measured and its definition [28,52]. For example, the NNT calculated for back pain relief cannot be applied to other outcome measures such as leg pain relief, and the NNT for pain relief of 50% or more will be different from that for pain relief of 30% or more. In addition, while a trial may report a primary outcome, it may also report numerous secondary outcomes of varying clinical importance, as well as results for ITT and PP populations. Therefore, multiple NNT estimates at different time points may be necessary to fully reflect the trial results over time, with the context and weight of each requiring careful consideration.

### 6.5. ITT and PP Populations

The patient population used for the NNT estimation is another factor that can influence the NNT value. For instance, we observed a variance in the NNT between ITT and PP populations. Furthermore, the definition of the ITT and PP populations is also worthy of note. In particular, the intention-to-treat analysis should evaluate outcomes in all patients according to the groups they were originally assigned by randomization, whether they received treatment or not [53]. However, this standard interpretation is not always applied. 

For example, one study in our review published an interim analysis of a partial study population [41], which formed the ITT group in our analysis; however, treatment outcomes in this subgroup may not reflect the true ITT study population. In addition, in the SUNBURST study by Deer et al. (2018), participants were randomized to t-SCS or burst stimulation after a positive response to a t-SCS trial (≥50% pain relief), with crossover after 12 weeks [40]. If a test is required prior to entering randomization, this is considered an enriched population, and the results cannot be generalized to the broader untested population. While the study (and our analysis) defined the ITT population as the randomized group, this population excluded trial failures, contrary to the intention-to-treat principle. The lack of a burst stimulation trial meant that a true ITT group could not be determined. Interpretation of the resulting NNT should bear this factor in mind, since the inclusion or exclusion of trial failures in a population affects the responder rate. Furthermore, the design of the SUNBURST study enriched the population for t-SCS responders, potentially favoring t-SCS response over burst stimulation [13], which may have contributed to the double-digit NNT estimate. The final example in our review is a study that allowed treatment crossover after 6 months; we could not estimate NNT values after this time due to the difficulty in determining the responder rate for the original ITT and PP populations [37]. In crossover trial designs, the NNT analysis requires outcome data for the initial treatment allocations.

We also noted in our analysis that it could be challenging to clarify ITT and PP population from the published data, and it is not always feasible to obtain additional information from corresponding authors. In one of our included studies, we found it difficult to confirm the actual ITT or PP treatment effects at 6 and 12 months since the reported responder rates were not identified as being derived from the ITT, modified ITT, or completer (PP) population. In addition, reverse calculations of the 3-month responder rate data yielded an unclear whole number of responders [39]. Therefore, we considered the included 12-month responder rate “tornado chart” in this case and performed a manual count of responders at 12 months. However, this approach would not be able to address the uncertainties in designating the subject as responder or non-responder when the percent pain reduction of subjects appears to be just under or at the 50% threshold [39].

### 6.6. Uncertainty

As with other estimates, the uncertainty in the NNT should be accompanied by an adequate CI. Furthermore, the CI calculation method should be specified [54]. If the effect size difference is statistically significant, the NNT 95% CI is straightforward to calculate and understand. However, when the effect size difference is not statistically significant, the NNT 95% CI is difficult to describe since it encompasses two regions (−∞ to lower boundary and upper boundary to ∞) [17]. Our review also highlighted that the granular level of data available in a study may influence the CI. For example, the number of responders and nonresponders within each group are necessary input values for the CI calculation. However, some trials in our review lacked this granular data and only reported total group numbers and responder rate. Consequently, the absolute patient numbers were reverse calculated, a method that may be prone to error.

### 6.7. Comparison Methodology

The NNT is best calculated from within-trial data generated from robust and well-designed controlled trials. Unfortunately, in our literature search, we found only 10 controlled trials over 50 years with usable responder rate data that compared SCS with either a standard treatment or another SCS modality for the treatment of axial back and/or leg pain [30,32,33,34,35,36,37,38,39,40,41,42]. We are aware of other controlled trials within the SCS sphere, for example, the ACCURATE study by Deer and colleagues, which compared DRG stimulation with t-SCS over 12 months in subjects with complex regional pain syndrome or causalgia. Unfortunately, we could not include this study in our review due to the predefined eligibility criteria. However, we estimated the 3- and 12-month NNT values for the ITT population as 4.47 (95% CI 2.68, 13.53) and 5.43 (95% CI 2.94, 34.66), respectively.

Interestingly, during our literature search, we noted that the SCS evidence base included a relatively large number of single-arm trials. Future work could make use of this rich data source to develop an indirect treatment comparison methodology. For example, averaged control arms for t-SCS and CMM populations could be generated by pooling responder rates across the single-arm trials. The NNT could subsequently be estimated for new SCS modalities using the averaged control arms.

### 6.8. Number Needed to Harm 

The potential benefits of a therapy must always be weighed against possible harms. The number needed to harm (NNH) is a complementary statistic that can also be useful in the NNT context. When comparing two treatments, the NNH tells us how many patients need to be treated (or exposed to a risk factor) with one treatment instead of the other for a given time before we expect one extra patient to incur a particular adverse event (AE) [18]. The NNH is calculated using the same principles as the NNT but is the reciprocal of the absolute risk increase (ARI).

While low NNT values are usually desirable, the opposite is true for the NNH. For example, consider a hypothetical trial of a new SCS therapy versus CMM that reported respective overall AE rates of 15% and 3% at the 12-month follow-up. In this case, the ARI is 12%, yielding an NNH of 8: if we treated eight patients with the new SCS therapy instead of CMM for 12 months, we would expect one extra patient to experience an AE. However, it may be more interesting to evaluate the NNH for the incidence of SCS-related AEs (which cannot occur in the CMM group). For example, a 10% incidence of SCS-related AEs would yield an NNH of 10, meaning that if 10 patients were treated with the new SCS therapy instead of CMM for 12 months, we would expect 1 of the SCS patients to experience an SCS-related AE. A higher incidence of SCS-related AEs would yield a lower NNH estimate.

In studies that compare SCS with CMM, the NNH for SCS-related AEs will always be positive (up to a maximum of infinity). In general, the NNH should be higher than the NNT to encounter positive outcomes more often than harmful ones [18]. However, in some cases, a low NNT, which may appear very promising, could be negated by a low NNH for a problematic AE [18]. In other trial scenarios, the NNH can be negative. For example, consider a trial of a new SCS therapy versus t-SCS that reported respective explant rates of 5% and 25%. In this scenario, the NNH would be −5. If the NNH 95% CI showed statistical significance, we would interpret the negative NNH as indicating that the patients given the new SCS therapy had a lower risk of explant over 12 months than those assigned to t-SCS. 

Unfortunately, in SCS trials, AE definition, grading, and reporting are often inconsistent or absent. Furthermore, many studies do not report granular AE data or explant rates. These factors make estimation and presentation of the NNH challenging, and may have overwhelmed this first presentation of SCS NNT estimates. Therefore, in a separate article, the concepts of NNH in the SCS field should be explored and proposed, along with guidelines on AE reporting.

### 6.9. Analysis and Limitations

Alongside more general contextual factors of the NNT, it is important to understand the NNT analysis performed and its limitations. We presented full details of our methodologies and are aware of several drawbacks. For example, we could not obtain clarifications from some study authors, particularly regarding patient numbers derived from available data. We also used the Wald method for the NNT 95% CI calculation. Although this approach is common, it has several deficiencies and may be less suitable for small sample sizes than the Wilson method [54]. In addition, during our literature search, we identified one other RCT comparing SCS treatment with a control arm for FBSS. However, the lack of response rate data excluded this study from our analysis [55].

## 7. Conclusions

Our analysis of historical controlled trials of SCS suggests that the NNT concept can be applied in this medical discipline and may support clinical decision-making, cost-effectiveness studies, and healthcare policymaking. As with other treatment modalities, it is important to consider the limitations of the NNT metric. Considering its sensitivity to multiple factors, we recommend its interpretation in the full context of response definition and control treatment used in the calculation. We also caution against comparisons across trials of different treatments since they are prone to interpretation biases.

## Figures and Tables

**Table 1:** Characteristics of included controlled trials.

Study Name/ID	Design	Single or Multicenter	Study Group(s)	Indication	Pain Relief Measure	Proportion with FBSS	Definition of Response
North 2005 [32]	RCT (open-label)	Single-center	t-SCS vs. Reoperation	Predominant leg pain	VAS	100%	≥50% pain relief + patient satisfaction
PROCESS: Kumar 2007 [30], Kumar 2008 [33]	RCT (open-label)	Multicenter	t-SCS vs. CMM	Predominant leg pain	VAS	100%	≥50% pain relief
Turner 2010 [42]	Controlled cohort (open-label)	Multicenter	t-SCS vs. PCM	Predominant leg pain	NRS	100%	≥50% pain relief
SENZA-RCT: Kapural 2015 [34], Kapural 2016 [35]	RCT (open-label)	Multicenter	10 kHz SCS vs. t-SCS	Back and leg pain	VAS	87%	≥50% pain relief without stimulation-related neurological deficit
SUNBURST: Deer 2018 [40]	RCOT (open-label)	Multicenter	Burst stim. vs. t-SCS	Trunk and/or limb pain	VAS	42%	≥50% pain relief *
SURF: Bolash 2019 [36]	RCT (open-label)	Multicenter	Externally powered 10 kHz SCS vs. 10–1500 Hz SCS	Back ± leg pain	VAS	100%	≥50% pain relief
PROMISE: Rigoard 2019 [37]	RCT (open-label)	Multicenter	t-SCS + OMM vs. CMM	Predominant back pain	NRS	100%	≥50% pain relief
WHISPER: North 2020 [41]	RCOT (open-label)	Multicenter	≤1.2 kHz subperc. SCS vs. t-SCS	Trunk and/or limb pain	VRS	46%	≥50% pain relief + no increase in pain medication intake
EVOKE: Mekhail 2020 [38]	RCT (double-blind)	Multicenter	Closed-loop SCS vs. t-SCS	Back and leg pain	VAS	60%	50% threshold + no increase in pain medication intake
Fishman 2021 [39]	RCT (open-label)	Multicenter	DTM SCS vs. t-SCS	Back and leg pain	VAS	59%	≥50% pain relief

CMM: conventional medical management; DTM: differential target multiplexed; FBSS: failed back surgery syndrome; NRS: Numerical Rating Scale; OMM: Optimal Medical Management; PCM: Pain Clinic Management; RCOT: randomized controlled crossover trial; RCT: randomized controlled trial; SCS: spinal cord stimulation; Subperc: subperception; t-SCS: traditional spinal cord stimulation; Stim: stimulation; VAS: visual analog score; VRS: visual rating scale. * The SUNBURST trial defined and reported postimplantation response as 30% pain relief; however, 50% pain relief data were also reported.

**Table 2:** NNT with 95% CI for SCS controlled trial ITT and PP populations.

Study/Article ID	Design	Treatment Group	Control Group	Follow-up Time	Pain Area	NNT for ITT Treatment Group vs. ITT Control Group (95% CI)	NNT for PP Treatment Group vs. PP Control Group (95% CI)
North 2005 [32]	RCT (open-label)	t-SCS	Reoperation	2.9 Yr	Comb. pain	5.00 (2.53, 250.00)	2.79 (1.63, 9.77)
PROCESS: Kumar 2007 [30]	RCT (open-label)	t-SCS	CMM	3 Mo	Leg pain	2.20 (1.64, 3.35)	2.13 (1.59, 3.25)
PROCESS: Kumar 2007 [30]	RCT (open-label)	t-SCS	CMM	6 Mo	Leg pain	2.64 (1.87, 4.51)	2.57 (1.81, 4.41)
PROCESS: Kumar 2007 [30]	RCT (open-label)	t-SCS	CMM	12 Mo	Leg pain	4.08 (2.58, 9.78)	3.74 (2.36, 9.09)
PROCESS: Kumar 2008 [33]	RCT (open-label)	t-SCS	CMM	24 Mo	Leg pain	3.27 (2.27, 5.80)	2.90 (2.03, 5.05)
Turner 2010 [42]	Controlled cohort (open-label)	t-SCS	PCM	6 Mo	Leg pain	7.99 (−3732.39, 3.99) †	8.08 (−384.11, 4.00) †
Turner 2010 [42]	Controlled cohort (open-label)	t-SCS	PCM	12 Mo	Leg pain	16.58 (−15.19, 5.36) †	15.24 (−14.19, 4.96) †
Turner 2010 [42]	Controlled cohort (open-label)	t-SCS	PCM	24 Mo	Leg pain	110.50 (−7.57, 6.66) †	63.57 (−6.82, 5.62) †
SENZA-RCT: Kapural 2015 [34]	RCT (open-label)	10 kHz SCS	t-SCS	3 Mo	Back pain	2.62 (1.96, 3.95)	2.47 (1.86, 3.67)
SENZA-RCT: Kapural 2015 [34]	RCT (open-label)	10 kHz SCS	t-SCS	3 Mo	Leg pain	3.58 (2.44, 6.77)	3.55 (2.41, 6.78)
SENZA-RCT: Kapural 2015 [34]	RCT (open-label)	10 kHz SCS	t-SCS	6 Mo	Back pain	4.08 (2.63, 9.02)	4.08 (2.59, 9.57)
SENZA-RCT: Kapural 2015 [34]	RCT (open-label)	10 kHz SCS	t-SCS	6 Mo	Leg pain	3.78 (2.52, 7.60)	3.77 (2.49, 7.77)
SENZA-RCT: Kapural 2015 [34]	RCT (open-label)	10 kHz SCS	t-SCS	12 Mo	Back pain	3.70 (2.48, 7.31)	3.66 (2.43, 7.42)
SENZA-RCT: Kapural 2015 [34]	RCT (open-label)	10 kHz SCS	t-SCS	12 Mo	Leg pain	3.70 (2.48, 7.31)	3.66 (2.43, 7.42)
SENZA-RCT: Kapural 2016 [35]	RCT (open-label)	10 kHz SCS	t-SCS	24 Mo	Back pain	3.54 (2.40, 6.71)	3.68 (2.39, 8.03)
SENZA-RCT: Kapural 2016 [35]	RCT (open-label)	10 kHz SCS	t-SCS	24 Mo	Leg pain	3.95 (2.58, 8.45)	4.23 (2.59, 11.54)
SUNBURST: Deer 2018 [40]	RCOT (open-label)	Burst stim.	t-SCS	3 Mo	Comb. pain	14.29 (−16.06, 4.94) †	13.71 (−15.81, 4.78) †
SURF: Bolash 2019 [36]	RCT (open-label)	Externally powered 10 kHz SCS	10–1500 Hz SCS	6 Mo	Back pain	7.78 (−16.83, 3.16) †	10.25 (−17.65, 3.97) †
PROMISE: Rigoard 2019 [37]	RCT (open-label)	t-SCS + OMM	OMM	6 Mo	Back pain	11.10 (6.04, 68.13)	8.70 (4.98, 34.47)
PROMISE: Rigoard 2019 [37]	RCT (open-label)	t-SCS + OMM	OMM	6 Mo	Leg pain	4.62 (3.16, 8.59)	3.67 (2.60, 6.24)
WHISPER: North 2019 [41]	RCOT (open-label)	≤1.2 kHz subperc. SCS	t-SCS	3 Mo	Comb. pain	10.00 (−17.99, 3.91) †	- *
EVOKE: Mekhail 2019 [38]	RCT (double-blind)	Closed-loop SCS	t-SCS	3 Mo	Comb. pain	5.15 (2.85, 26.66)	6.16 (3.23, 67.14)
EVOKE: Mekhail 2019 [38]	RCT (double-blind)	Closed-loop SCS	t-SCS	3 Mo	Back pain	4.79 (2.72, 19.81)	5.47 (2.97, 34.51)
EVOKE: Mekhail 2019 [38]	RCT (double-blind)	Closed-loop SCS	t-SCS	3 Mo	Leg pain	9.57 (−19.78, 3.85) †	19.71 (−11.50, 5.31) †
EVOKE: Mekhail 2019 [38]	RCT (double-blind)	Closed-loop SCS	t-SCS	12 Mo	Comb. pain	5.15 (2.83, 29.17)	7.10 (−148.78, 3.47) †
EVOKE: Mekhail 2019 [38]	RCT (double-blind)	Closed-loop SCS	t-SCS	12 Mo	Back pain	5.15 (2.81, 31.50)	6.84 (−79.46, 3.28) †
EVOKE: Mekhail 2019 [38]	RCT (double-blind)	Closed-loop SCS	t-SCS	12 Mo	Leg pain	5.15 (2.83, 29.17)	7.10 (−148.78, 3.47) †
Fishman 2021 [39]	RCT (open-label)	DTM SCS	t-SCS	12 Mo	Back pain	6.24 (−98.36, 3.02) †	4.25 (2.36, 21.37)

CI: confidence interval; CMM: conventional medical management; Comb: combined; DTM: differential target multiplexed; ITT: intention-to-treat; Mo: months; N/A: not applicable; NNT: number needed to treat; OMM: Optimal Medical Management; PCM: Pain Clinic Management; PP: per-protocol; RCOT: randomized controlled crossover trial; RCT: randomized controlled trial; SCS: spinal cord stimulation; Subperc: subperception; t-SCS: traditional spinal cord stimulation; Stim: stimulation; Yr: years. † Discontinuous 95% CI: (−∞ to lower boundary) ∪ (upper boundary to ∞), i.e., the difference between the treatments is not statistically significant for the given outcome and time frame. * Interim data analysis.

## Data Availability

All the data generated during the review are included in the illustrations.

## Supplementary Materials

The supplementary materials are available online at https://www.mdpi.com/2227-9059/10/2/497/s1, Table S1: Data extracted from controlled trials for the ITT and PP populations.
