# Supplementary material for: Concept of the Number Needed to Treat for the Analysis of Pain Relief Outcomes in Patients Treated with Spinal Cord Stimulation
Source: Biomedicines. 2022 Feb 20;10(2):497. doi: 10.3390/biomedicines10020497 (PMC8962384; doi:10.3390/biomedicines10020497)
Supplement: Supplementary file 1 [file biomedicines-10-00497-s001.zip › biomedicines-1536628-supplementary.pdf]

Supplementary Information

Table S1. *Data extracted from controlled trials for the ITT and PP populations.*

Study/Article ID	***Design***	***Treatment group (TG)***	***Control group (CG)***	***Follow-up time***	***Pain area***	***Responder rate/Total N/# Responders/# Nonresponders*** ***ITT population***	***PP population***
North 2005 (32)	*RCT (open-label)*	*t-SCS*	*Reoperation*	*2.9 Yr*	*Comb. pain*	*TG: 30.0%/30/9/21 CG: 10.0%/30/3/27*	*TG: 47.4%/19/9/10 CG: 11.5%/26/3/23*
PROCESS: Kumar 2007 (30)	*RCT (open-label)*	*t-SCS*	*CMM*	*3 Mo*	*Leg pain*	*TG: 53.8%/52/28/24 CG: 8.3%/48/4/44*	*TG: 56.0%/50/28/22 CG: 9.1%/44/4/40*
PROCESS: Kumar 2007 (30)	*RCT (open-label)*	*t-SCS*	*CMM*	*6 Mo*	*Leg pain*	*TG: 46.2%/52/24/28 CG: 8.3%/48/4/44*	*TG: 48.0%/50/24/26 CG: 9.1%/44/4/40*
PROCESS: Kumar 2007 (30)	*RCT (open-label)*	*t-SCS*	*CMM*	*12 Mo*	*Leg pain*	*TG: 30.8%/52/16/36 CG: 6.3%/48/3/45*	*TG: 34.0%/47/16/31 CG: 7.3%/41/3/38*
PROCESS: Kumar 2008 (33)	*RCT (open-label)*	*t-SCS*	*CMM*	*24 Mo*	*Leg pain*	*TG: 32.7%/52/17/35 CG: 2.1%/48/1/47*	*TG: 37.0%/46/17/29 CG: 2.4%/41/1/40*
Turner 2010 (42)	*Controlled cohort (open-label)*	*t-SCS*	*PCM*	*6 Mo*	*Leg pain*	*TG: 17.6%/51/9/42 CG: 5.1%/39/2/37*	*TG: 17.6%/51/9/42 CG: 5.3%/38/2/36*
Turner 2010 (42)	*Controlled cohort (open-label)*	*t-SCS*	*PCM*	*12 Mo*	*Leg pain*	*TG: 13.7%/51/7/44 CG: 7.7%/39/3/36*	*TG: 14.9%/47/7/40 CG: 8.3%/36/3/33*
Turner 2010 (42)	*Controlled cohort (open-label)*	*t-SCS*	*PCM*	*24 Mo*	*Leg pain*	*TG: 13.7%/51/7/44 CG: 12.8%/39/5/34*	*TG: 16.3%/43/7/36 CG: 14.7%/34/5/29*
SENZA-RCT: Kapural 2015 (34)	*RCT (open-label)*	*10 kHz SCS*	*t-SCS*	*3 Mo*	*Back pain*	*TG: 74.3%/101/75/26 CG: 36.1%/97/35/62*	*TG: 84.3%/89/75/14 CG: 43.8%/80/35/45*
SENZA-RCT: Kapural 2015 (34)	*RCT (open-label)*	*10 kHz SCS*	*t-SCS*	*3 Mo*	*Leg pain*	*TG: 73.3%/101/74/27 CG: 45.4%/97/44/53*	*TG: 83.1%/89/74/15 CG: 55.0%/80/44/36*
SENZA-RCT: Kapural 2015 (34)	*RCT (open-label)*	*10 kHz SCS*	*t-SCS*	*6 Mo*	*Back pain*	*TG: 67.3%/101/68/33 CG: 42.8%/97/42/55*	*TG: 76.4%/89/68/21 CG: 51.9%/80/42/38*
SENZA-RCT: Kapural 2015 (34)	*RCT (open-label)*	*10 kHz SCS*	*t-SCS*	*6 Mo*	*Leg pain*	*TG: 71.3%/101/72/29 CG: 44.9%/97/44/53*	*TG: 80.9%/89/72/17 CG: 54.4%/80/44/36*
SENZA-RCT: Kapural 2015 (34)	*RCT (open-label)*	*10 kHz SCS*	*t-SCS*	*12 Mo*	*Back pain*	*TG: 69.3%/101/70/31 CG: 42.3%/97/41/56*	*TG: 78.7%/89/70/19 CG: 51.3%/80/41/39*

SENZA-RCT: Kapural 2015 (34)	*RCT (open-label)*	*10 kHz SCS*	*t-SCS*	*12 Mo*	*Leg pain*	*TG: 69.3%/101/70/31*<br>*CG: 42.3%/97/41/56*	*TG: 78.7%/89/70/19*<br>*CG: 51.3%/80/41/39*
SENZA-RCT: Kapural 2016 (35)	*RCT (open-label)*	*10 kHz SCS*	*t-SCS*	*24 Mo*	*Back pain*	*TG: 64.4%/101/65/36*<br>*CG: 36.1%/97/35/62*	*TG: 76.5%/85/65/20*<br>*CG: 49.3%/71/35/36*
SENZA-RCT: Kapural 2016 (35)	*RCT (open-label)*	*10 kHz SCS*	*t-SCS*	*24 Mo*	*Leg pain*	*TG: 61.4%/101/62/39*<br>*CG: 36.1%/97/35/62*	*TG: 72.9%/85/62/23*<br>*CG: 49.3%/71/35/36*
SUNBURST: Deer 2018 (40)	*RCOT (open-label)*	*Burst stim.*	*t-SCS*	*3 Mo*	*Comb. pain*	*TG: 39.0%/100/39/61*<br>*CG: 32.0%/100/32/68*	*TG: 40.6%/96/39/57*<br>*CG: 33.3%/96/32/64*
SURF: Bolash 2019 (36)	*RCT (open-label)*	*Externally powered 10 kHz SCS*	*10-1500 Hz SCS*	*6 Mo*	*Back pain*	*TG: 70.0%/50/35/15*<br>*CG: 57.1%/49/28/21*	*TG: 92.1%/38/35/3*<br>*CG: 82.4%/34/28/6*
PROMISE: Rigoard 2019 (37)	*RCT (open-label)*	*t-SCS + OMM*	*OMM*	*6 Mo*	*Back pain*	*TG: 13.6%/110/15/95*<br>*CG: 4.6%/108/5/103*	*TG: 16.3%/92/15/77*<br>*CG: 4.8%/104/5/99*
PROMISE: Rigoard 2019 (37)	*RCT (open-label)*	*t-SCS + OMM*	*OMM*	*6 Mo*	*Leg pain*	*TG: 30.0%/110/33/77*<br>*CG: 8.3%/108/9/99*	*TG: 35.9%/92/33/59*<br>*CG: 8.7%/104/9/95*
WHISPER: North 2019 (41)	*RCOT (open-label)*	*≤1.2 kHz subperc. SCS*	*t-SCS*	*3 Mo*	*Comb. pain*	*TG: 38.6%/70/27/43*<br>*CG: 28.6%/70/20/50*	*N/A**
EVOKE: Mekhail 2019 (38)	*RCT (double-blind)*	*Closed-loop SCS*	*t-SCS*	*3 Mo*	*Comb. pain*	*TG: 76.1%/67/51/16*<br>*CG: 56.7%/67/38/29*	*TG: 87.9%/58/51/7*<br>*CG: 71.7%/53/38/15*
EVOKE: Mekhail 2019 (38)	*RCT (double-blind)*	*Closed-loop SCS*	*t-SCS*	*3 Mo*	*Back pain*	*TG: 74.6%/67/50/17*<br>*CG: 53.7%/67/36/31*	*TG: 86.2%/58/50/8*<br>*CG: 67.9%/53/36/17*
EVOKE: Mekhail 2019 (38)	*RCT (double-blind)*	*Closed-loop SCS*	*t-SCS*	*3 Mo*	*Leg pain*	*TG: 74.6%/67/50/17*<br>*CG: 64.2%/67/43/24*	*TG: 86.2%/58/50/8*<br>*CG: 81.1%/53/43/10*
EVOKE: Mekhail 2019 (38)	*RCT (double-blind)*	*Closed-loop SCS*	*t-SCS*	*12 Mo*	*Comb. pain*	*TG: 73.1%/67/49/18*<br>*CG: 53.7%/67/36/31*	*TG: 89.1%/55/49/6*<br>*CG: 75.0%/48/36/12*
EVOKE: Mekhail 2019 (38)	*RCT (double-blind)*	*Closed-loop SCS*	*t-SCS*	*12 Mo*	*Back pain*	*TG: 70.1%/67/47/20*<br>*CG: 50.7%/67/34/33*	*TG: 85.5%/55/47/8*<br>*CG: 70.8%/48/34/14*
EVOKE: Mekhail 2019 (38)	*RCT (double-blind)*	*Closed-loop SCS*	*t-SCS*	*12 Mo*	*Leg pain*	*TG: 73.1%/67/49/18*<br>*CG: 53.7%/67/36/31*	*TG: 89.1%/55/49/6*<br>*CG: 75.0%/48/36/12*
Fishman 2021 (39)	*RCT (open-label)*	*DTM SCS*	*t-SCS*	*12 Mo*	*Back pain*	*TG: 53.7%/67/36/31*<br>*CG: 37.7%/61/23/38*	*TG: 85.7%/42/36/6*<br>*CG: 62.2%/37/23/14*

CMM: Conventional Medical Management; Comb: Combined; Conf: Conference; DTM: Differential Target Multiplexed; ITT: Intention-to-treat; Mo: Months; N/A: Not applicable; OMM: Optimal Medical

Management; PP: Per-protocol; RCOT: Randomized Controlled Crossover Trial; RCT: Randomized Controlled Trial; SCS: Spinal cord stimulation; Stim: Stimulation; Subperc: Subperception; t-SCS: Traditional Spinal Cord Stimulation; Yr: Years.

** Interim data analysis.*
